# PSmad3+/Olig2− expression defines a subpopulation of *gfap-*GFP+/Sox9+ neural progenitors and radial glia-like cells in mouse dentate gyrus through embryonic and postnatal development

**DOI:** 10.3389/fnins.2023.1204012

**Published:** 2023-09-18

**Authors:** Kyoji Ohyama, Hiroshi M. Shinohara, Shoichiro Omura, Tomomi Kawachi, Toru Sato, Keiko Toda

**Affiliations:** Department of Histology and Neuroanatomy, Tokyo Medical University, Tokyo, Japan

**Keywords:** Smad3, GFAP, Olig2, SGZ, radial glia-like cells, astrocytes, dentate gyrus

## Abstract

In mouse dentate gyrus, radial glia-like cells (RGLs) persist throughout life and play a critical role in the generation of granule neurons. A large body of evidence has shown that the combinatorial expression of transcription factors (TFs) defines cell types in the developing central nervous system (CNS). As yet, the identification of specific TFs that exclusively define RGLs in the developing mouse dentate gyrus (DG) remains elusive. Here we show that phospho-Smad3 (PSmad3) is expressed in a subpopulation of neural progenitors in the DG. During embryonic stage (E14-15), PSmad3 was predominantly expressed in *gfap*-GFP-positive (GFP+)/Sox2+ progenitors located at the lower dentate notch (LDN). As the development proceeds (E16-17), the vast majority of PSmad3+ cells were GFP+/Sox2+/Prox1_low_+/Ki67+ proliferative progenitors that eventually differentiated into granule neurons. During postnatal stage (P1–P6) PSmad3 expression was observed in GFP+ progenitors and astrocytes. Subsequently, at P14–P60, PSmad3 expression was found both in GFP^+^ RGLs in the subgranular zone (SGZ) and astrocytes in the molecular layer (ML) and hilus. Notably, PSmad3+ SGZ cells did not express proliferation markers such as PCNA and phospho-vimentin, suggesting that they are predominantly quiescent from P14 onwards. Significantly PSmad3+/GFP+ astrocytes, but not SGZ cells, co-expressed Olig2 and S100β. Together, PSmad3+/Olig2− expression serves as an exclusive marker for a specific subpopulation of GFP+ neural progenitors and RGLs in the mouse DG during both embryonic and postnatal period.

## Introduction

Using *gfap-*GFP transgenic mice expressing GFP under the control of mouse *gfap* promoter, it has previously been shown that *gfap-*GFP*+* (GFP+) progenitors around the dentate notch (DN) contribute to granule neurons in the dentate gyrus (DG) during development (Altman and Bayer, 1990; Seki et al., 2014). The GFP+ progenitors have also been suggested to contribute to other cell types: radial glia-like cells (RGLs) and protoplasmic astrocytes (Brunne et al., 2013). After birth, neurogenesis takes place mainly in the hilus and its border from the granule cell layer (GCL), becoming confined to the subgranular zone (SGZ) by postnatal day 14 (P14; Namba et al., 2005; Seki et al., 2014). Some GFP+ progenitors are converted to adult type RGLs immediately after birth (Matsue et al., 2017). However, the mechanism by which RGLs are specified during DG development is largely unknown.

There is considerable evidence that combinatorial expressions of TFs under the influence of signaling molecules define progenitor cell types in the developing CNS. However, it is currently unknown what TF code distinguishes RGLs from astrocytes, both of which appear mostly during the early postnatal period. Molecular markers for RGLs such as Sox9 are important to maintain RGLs in adulthood. However, they are also expressed in astrocytes, making it difficult to understand the mechanism by which RGLs and astrocytes develop in different ways.

Previous studies have shown that varieties of signals control the development of the dentate gyrus (DG) both in the embryo and the adult. Both BMP7 and Wnt signals regulate the expression of transcription factor Prox1, thereby governing the fate of granule neurons in the DG (Galceran et al., 2000; Choe et al., 2013). Sox2-dependent Shh signaling plays a key role both in the development of RGLs and sustained neurogenesis at the SGZ in adult mice (Li et al., 2013). ALK5-dependent TGFβ signaling through pSmad2 maintains late events during adult hippocampal neurogenesis (He et al., 2014). Smad3 is crucial for neuronal survival and adult neurogenesis in the hippocampus (Tapia-González et al., 2013). However, lack of TGFβ-Smad signaling does not affect the development of dentate granule neurons (Choe et al., 2013). These studies suggest that TGFβ-Smad2/3 signaling is essential for the development of adult but not embryonic granule neurons in the hippocampus. However, it remains unclear whether TGFβ-Smad signaling plays a role for other cell types that derived from GFP+ progenitors, namely astrocytes in the developing DG.

Phospho-Smad3 (PSmad3) has been shown to regulate not only progenitor specification and neuronal differentiation in the embryonic spinal cord (García-Campmany and Martí, 2007). As yet its expression pattern in the developing mouse DG has not been well studied. A recent study pointed out that TGFβ-PSmad3 pathway controls the glioblastoma stemness (Ikushima et al., 2009). Given the migratory and proliferative properties of the GFP+ DG progenitors (Seki et al., 2014), they may share some properties. In this study, we therefore examined the expression patterns of PSmad3 in the developing mouse DG. We show that PSmad3 is expressed in both a subpopulation of GFP+/Sox9+ progenitors, RGLs, and astrocytes throughout the embryonic and postnatal period. It is worthy of note that Olig2 expression distinguishes astrocytes from the RGLs. Together, PSmad3+/Olig2− expression defines a subpopulation of GFP+/Sox9+ neural progenitors and RGLs at the SGZ in both embryonic and postnatal mouse DG.

## Materials and methods

### Animals

*Gfap-*GFP transgenic mice that express GFP under the control of mouse *gfap* promoter (Suzuki et al., 2003) were housed under a standard condition (12-h light/dark cycle) in the animal care facility of Tokyo Medical University. All experiments were carried out in accordance with the guideline of the Institutional Animal Care and Use Committees and conform to the National Institutes of Health Guide for the Care and Use of Laboratory Animals (NIH Publication No. 80-23) revised in 1996. All efforts were made to minimize the number of animals used and their suffering. Embryos and pups of the *gfap*-GFP transgenic mice above were used. The day on which a vaginal plug was found was designated as embryonic day 0.5 (E0.5), and the day of birth was designated as postnatal day 0.5 (P0.5).

### Tissue preparation

Embryos were harvested at E14.5−E18.5, and postnatal mice were anesthetized and perfused with 4% paraformaldehyde (PFA) in 0.1 M phosphate buffer (PB), pH7.4, at room temperature. The brains were removed and washed with PBS and immersed in 30% sucrose/0.1 M PB. The forebrains were embedded into OCT compound and stored at –70°C. Cryosections were cut at 25 μm thickness.

### Antibodies

Antibodies used in this study are in the followings: mouse anti-E-cad (BD Transduction Laboratories 61081, 1:250); chick anti-GFAP (Millipore, 1:5,000); chick anti-GFP IgY (Abcam ab13970, 1:5,000); rabbit anti-GLAST polyclonal antibody (Covalab (France) PA-INS, 1:1,000); rabbit anti-Ki67 polyclonal antibody (Novocastra, NCL-Ki67p, 1:1,000); goat anti-NeuroD antibody (N-19; Santa Cruz, sc-1,084, 1:1,000); rabbit anti-Olig2 IgG (Millipore, AB9610, 1:1,000); goat anti-Olig2 (R&D systems, 1:1,000); mouse anti-PCNA (Novocastra, PC-10, 1:100); rabbit anti-PSmad3(S423/S425) polyclonal antibody (Millipore 07-1389, 1:1,000); mouse anti-phospho-vimentin IgG (MBL, D076-3S, 1:1,000); goat anti-Prox1 IgG (R&D systems, 1:1,000); mouse anti-S100β (Sigma, 1:1,000); rabbit Sox2 antibody (Millipore, 1:1,000); goat anti-Sox2 IgG (R&D systems, 1:1,000); goat anti-Sox9 polyclonal antibody (R&D systems, 1:1,000).

### Immunohistochemistry

Cryosections were processed for immunohistochemistry as described previously (Ohyama et al., 2005; Seki et al., 2014). Briefly, cryosection of the hippocampus were incubated with primary antibodies overnight at 4°C. For some antibody labeling experiments (Ki67, phospho-vimentin), antigen retrieval with Histo VT One (Nacalai, Japan) was carried out following manufacturer’s instructions. After wash with PBS three times, the sections were incubated with secondary antibodies for 45 min at room temperature. After wash with PBS three times, the sections were mounted with Vectashield (Vector lab). Images were taken with a Zeiss confocal microscope LSM700. In some cases, fluorescence images were digitally zoomed at 0.5x to 2x. Stacks of optical sections (1.8 μm in thickness/optical section) were obtained at 0.9 μm increments on the z-axis using x20 objective. The images were corrected for brightness and contrast and composed using Zeiss Image Browser, ZEN software (Zeiss, Thomwood, NY) and Adobe Photoshop CS6 (San Jose, CA). Mice (*n* = 3–6) were examined for individual experiments, and, for quantification of some experiments, 9 sections at least were analyzed for each using Fiji of image J. Mean ± SE was indicated in the results.

## Results

### *Gfap-*GFP+ cells contribute to RGLs at the SGZ and astrocytes in the molecular layer and hilus

**Figure 1:**
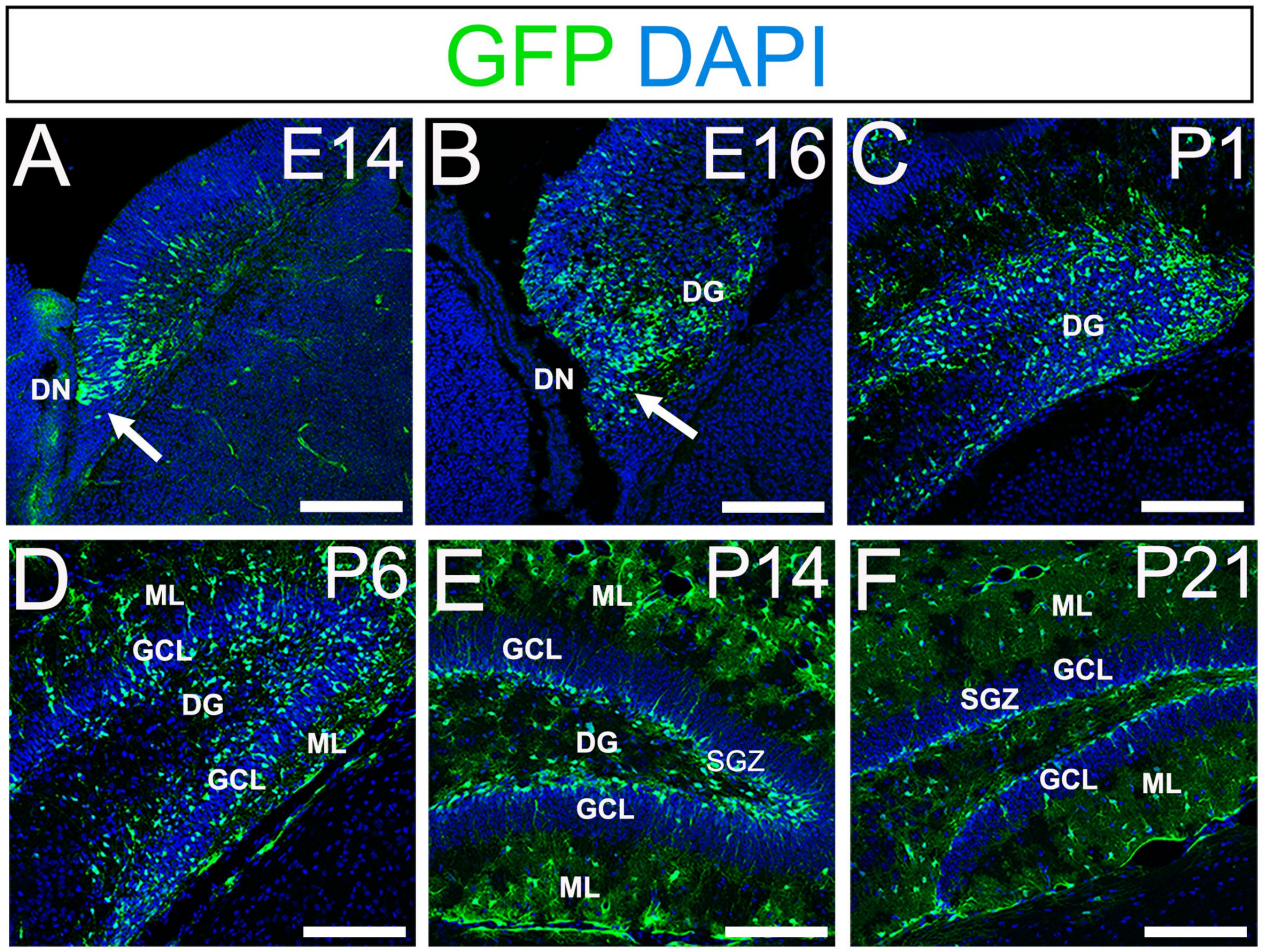
*Gfap-*GFP*+* (GFP+) progenitors contribute to RGLs and astrocytes in developing mouse DG **(A,B)** GFP+ cells are found at the DN and forming DG at E14 and E16. **(C,D)** Many GFP+ cells are observed in the developing DG at P1 and P6. **(E,F)** GFP+ cells are located at SGZ and ML of the DG at P14-21. DN, dentate notch; DG, dentate gyrus; GCL, granule cell layer; ML, molecular layer; SGZ, subgranular zone. Scale bars: all 200 μm.

Using *gfap-*GFP mice (Seki et al., 2014; Matsue et al., 2017), we first monitored the contributions of *gfap-*GFP-positive (GFP+) progenitors in the developing mouse dentate gyrus (DG). GFP+ progenitors were found in the ventricular zone (VZ) around the dentate notch (DN) and in the primordium of the DG at E14–E16 (Figures 1A,B). At postnatal day1 (P1), many GFP+ progenitors accumulate in the developing DG (Figure 1C). By P6 hilus, granule cell layer (GCL), and molecular layer (ML) become apparent (Figure 1D). GFP+ cells were observed not only in the hilus of the DG, but also in the ML. At P14-21, in addition to the GFP+ astrocytes in the hilus and ML, GFP+ cells were also found at the SGZ where RGLs reside postnatally (Figures 1E,F). Taken together, our data indicate that GFP+ progenitors contribute to both RGLs at the SGZ and astrocytes in the hilus and ML over time (Supplementary Figure 1).

### PSmad3 expression in GFP+ RGLs in early developing mouse DG

**Figure 2:**
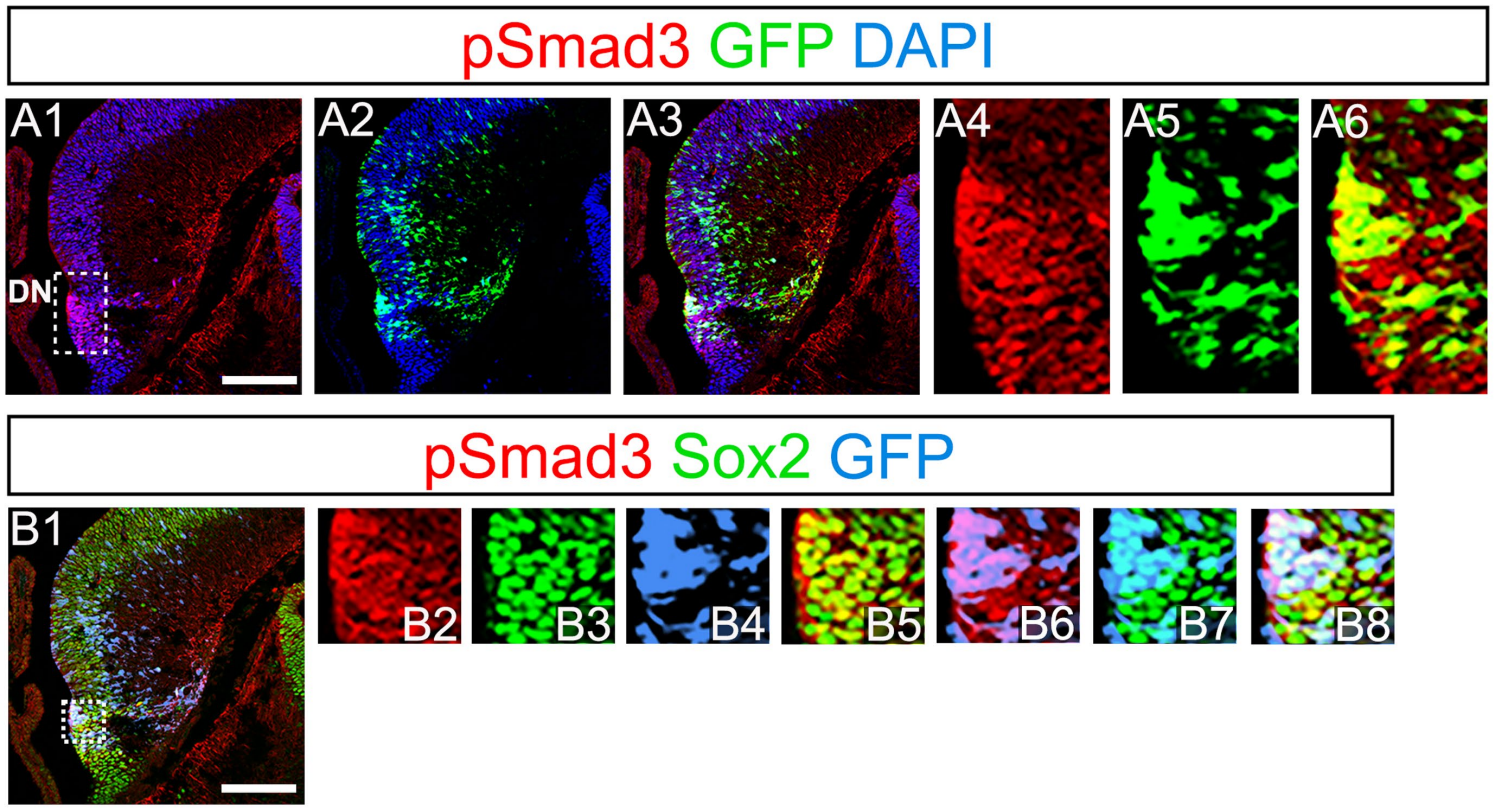
PSmad3 expression in GFP*+*/Sox2+ cells at the lower DN and their migratory stream toward developing DG **(A1–A6)** PSmad3 expression was found in GFP+ cells at the lower DN and their migratory stream toward the DG primordium at E14. **(B1–B8)** Co-expression of pSmad3, Sox2, and GFP+ at E14. Box in panels **(A1,B1)** indicates region shown in panels (**A4-A6**,**B2–B8**, respectively). Scale bars: all 200 μm. PSmad3 is expressed in GFP+/Sox2+ cells at E14 **(A1–A6)** PSmad3 is expressed in GFP+ cells. **(B1–B8)** Many PSmad3+/GFP+ cells co-express Sox2. Box in panels **(A1,B1)** indicates region shown in panels (**A4–A6,B2–B8**, respectively).

PSmad3 has been shown to control the proliferative and migratory behavior of glioblastoma (Ikushima et al., 2009). GFP+/Sox2+ neural progenitors also possess both proliferative and migratory properties (Seki et al., 2014). This led us to examine whether PSmad3 is expressed in the GFP+/Sox2+ neural progenitors in developing mouse DG. At E14, PSmad3 expression was found in the ventricular zone (VZ) of the lower part of the DN and in a migrating stream of GFP+/Sox2+ progenitors toward the DG primordium (*n* = 3; Figures 2A1−B8).

**Figure 3:**
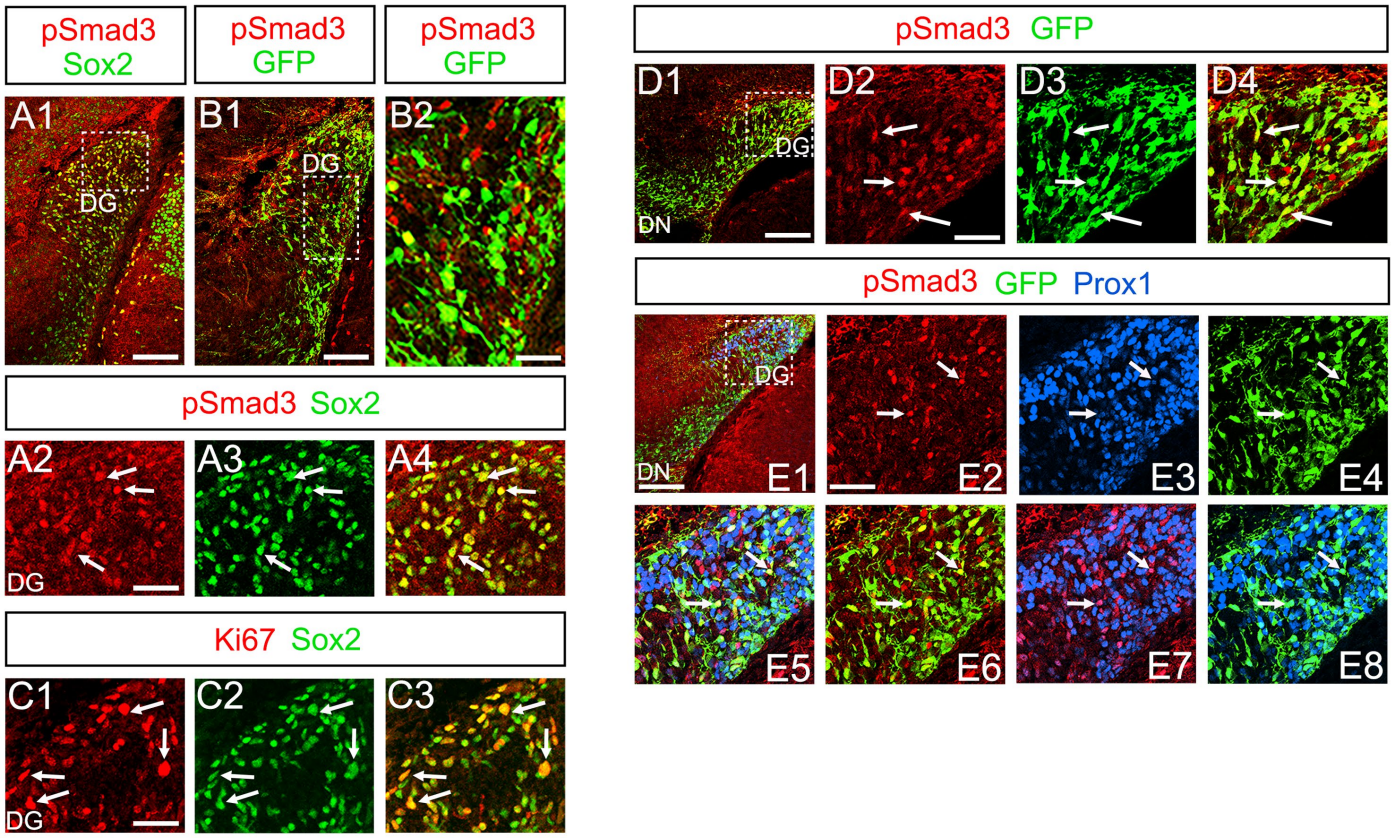
PSmad3+/GFP+ cells are Ki67+ proliferative RGLs and co-express Prox1_Low_ in embryonic DG **(A1–A4)** PSmad3 co-express Sox2 at E16. **(B1,B2)** PSmad3+ cells co-express GFP at E17. **(C1–C3)** 44% of Sox2+ cells were Ki67+ proliferating cells. **(D1–D4)** PSmad3+/ GFP+ cells observed at E17. **(E1–E8)** PSmad3 is expressed in GFP+/Prox1_low_ + cells at E18 (arrows). Scale bars: 200 μm in **A1,B1,D1,E1**; 50 μm in **A2–A4,B2,C1–C3,D2–D4,E2–E8**.

At E16-P3, 64.4% ± 6.4% of Sox2+ cells co-expressed PSmad3 in the DG (*n* = 4), and 44.3% ± 3.4% of Sox2+ cells were Ki67+ proliferative cells (Figures 3A1–A4,C1–C3; *n* = 4). PSmad3 and GFP were co-expressed in dentate progenitors. 32.5% ± 3.6% of PSmad3+ cells were Prox1_low_ + before birth (33% ± 3%, *n* = 5; Figures 3B1,B2,D1–D4,E1–E8). Given that GFP+ progenitors give rise to Prox1+ granule neurons (Seki et al., 2014), these data suggest that PSmad3 is expressed in a subpopulation of GFP+ progenitors that give rise to granule neurons.

### PSmad3 expression in GFP+ RGLs and astrocytes in the postnatal DG

**Figure 4:**
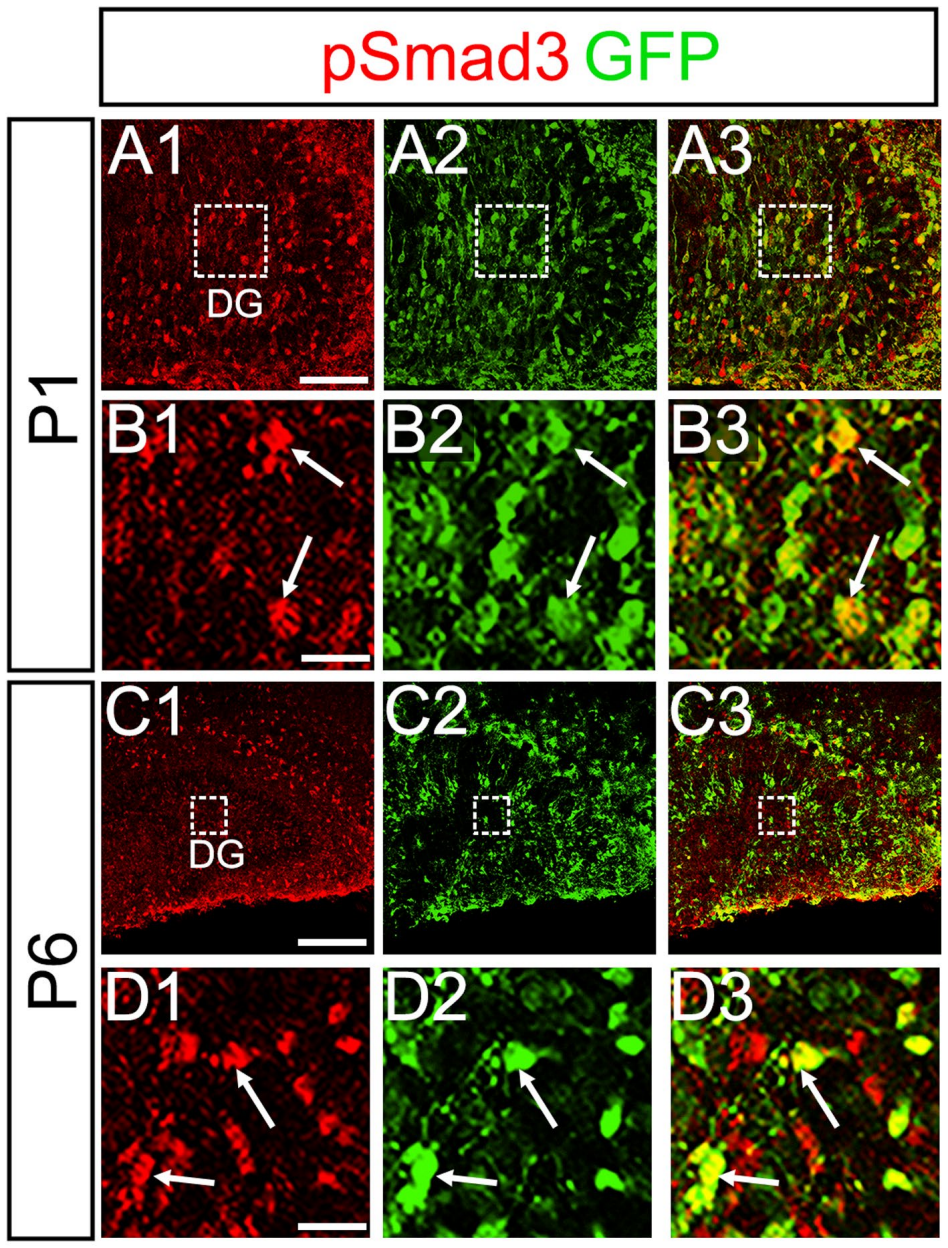
PSmad3 is expressed in GFP+ RGLs in early postnatal DG **(A1–D3)** PSmad3 expression in GFP+ RGLs at P1 and P6 (arrows in **B1–B3,D1–D3**). Box in panels (**A1–A3,C1–C3**) indicates region shown in panels (**B1–B3,D1–D3**). Scale bars: 200 μm in **A1–A3,C1–C3**; 50 μm in **B1–B3,D1–D3**.

**Figure 5:**
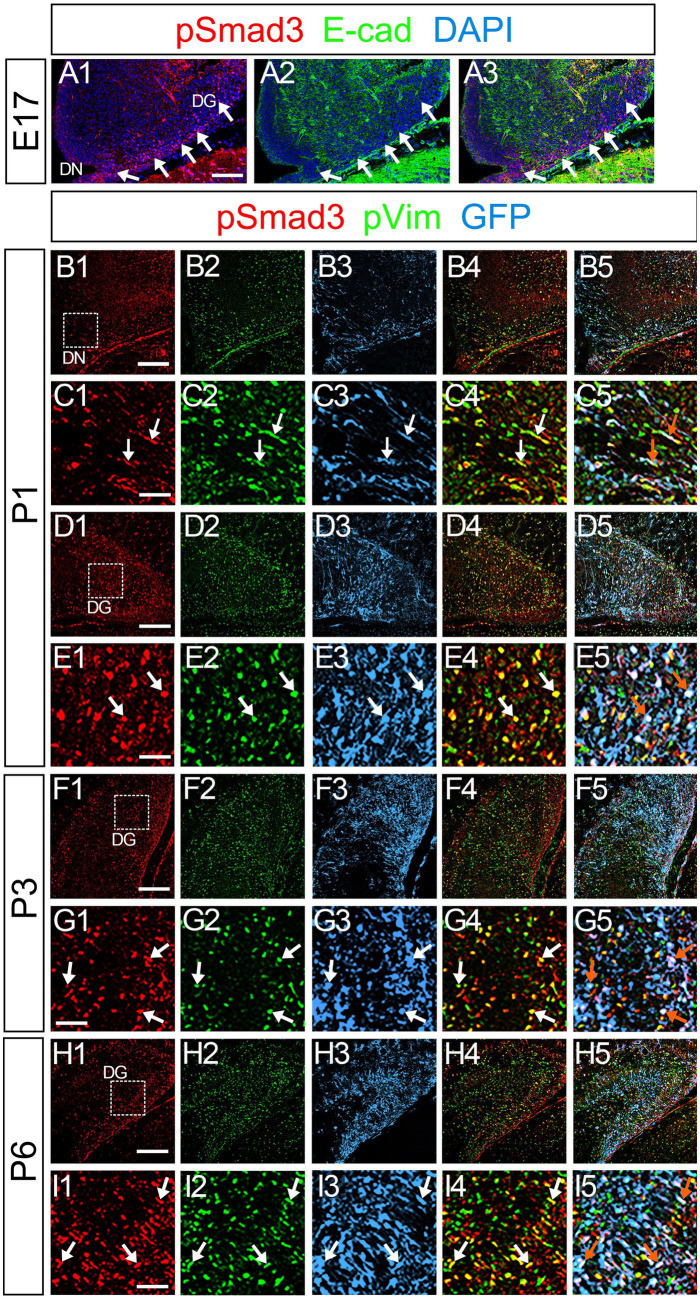
PSmad3+/GFP+ cells are pVim+ proliferating basal RGLs in postnatal DG **(A1–A3)** PSmad3 expression is abundant in E-Cad-negative area of the DG at E17. **(B1–I5)** PSmad3+/GFP+ cells co-express a proliferative basal RGLs marker pVim at DN and DG at P1–P6 (arrows in **C1–C5,E1–E5,G1–G5,I1–I5**). Box in panels (**B1,D1,F1,H1**) indicates region shown in panels (**C1–C5,E1–E5,G1–G5,I1–I5**, respectively). Scale bars: 200 μm in **A1–A3,B1–B5,D1–D5,F1–F5,H1–H5**; 100 μm in **C1–C5,E1–E5,G1–G5,I1–I5**.

PSmad3 was also expressed in GFP+ RGLs at P1–P6 (45.4% ± 2.7%, *n* = 5; Figures 4A1–D3). At P1 GFP+ cells co-expressed GLAST, another marker for RGLs in the DG (Supplementary Figures 2A1–A6). Given that PSmad3 is a regulator of epithelial mesenchymal transition (EMT), and that an EMT-like mechanism operates in the developing CNS (Itoh et al., 2013; Singh and Solecki, 2015; Singh et al., 2016), we compared an expression pattern of PSmad3 with that of E cadherin (E-cad), a marker for epithelial cells. PSmad3+ cells were enriched alongside the pial surface of the DG where E-cad was less abundant (Figures 5A1–A3). Instead, vimentin expression was prominent in the DG (data not shown). Moreover, at P1–P6 the PSmad3+/GFP+ cells were found to co-express phospho-vimentin (pVim), a marker for proliferative RGLs both in the DN and DG (*n* = 3; Figures 5B1–I5). pVim expression was also found in Sox2+ cells at P6, and some pVim+ nuclei highlighted a mitotic feature of PSmad3+ RGLs (data not shown). PSmad3+/GFP+ cells were PCNA+ at P3–P6 (*n* = 3, Supplementary Figures 3A1–B6). Consistent with this, Ki67 expression was also found in the GFP+ RGLs (28 ± 5%, *n* = 6; Supplementary Figures 4A1–A6).

**Figure 6:**
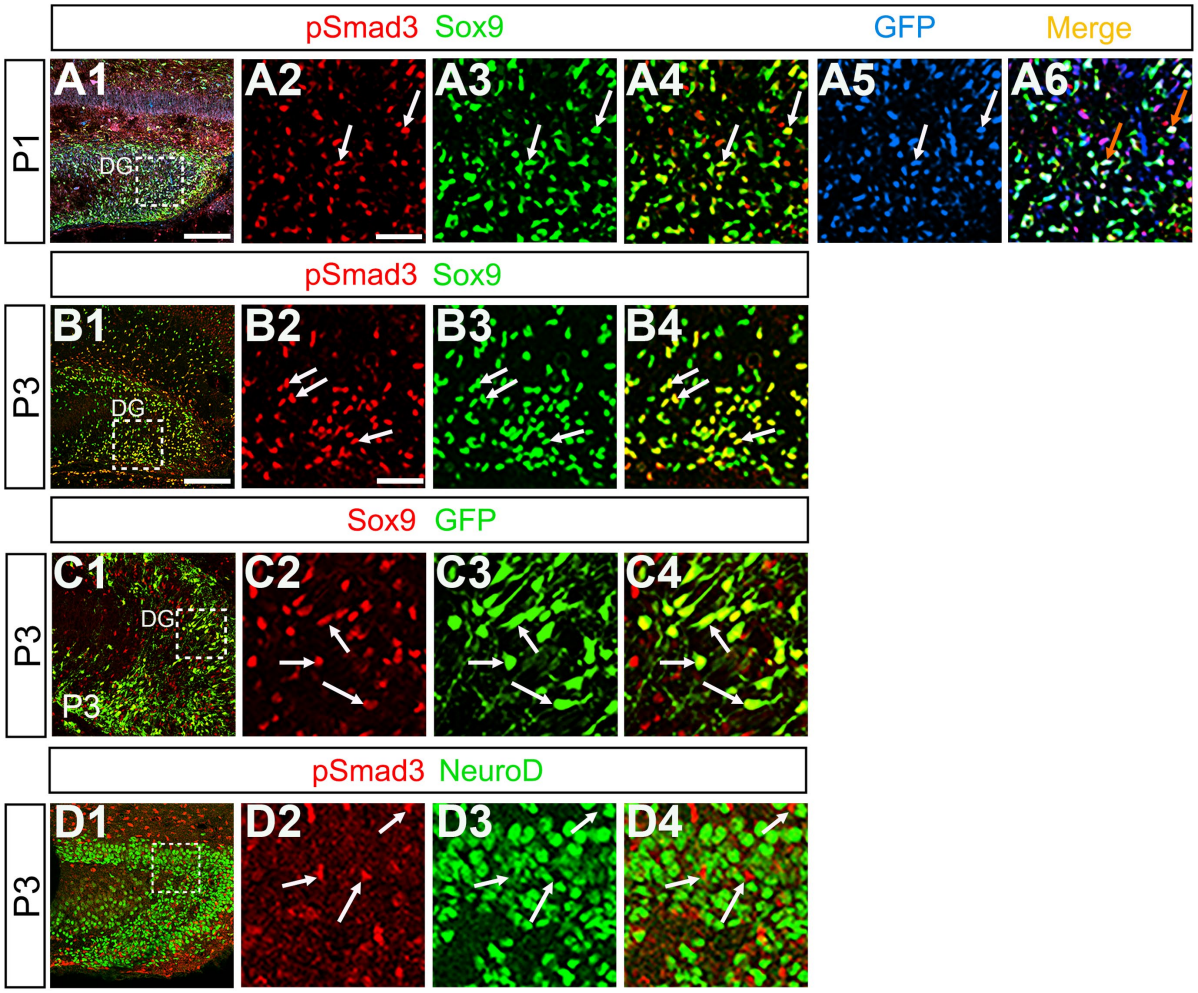
PSmad3+/GFP+ RGLs express Sox9 but not NeuroD **(A1–A6)** PSmad3 + cells co-express Sox9 in DG at P1 (arrows in **A2–A4**). The PSmad3+/Sox9+ cells also co-express GFP (arrows in **A5,A6**). **(B1–B4)** Co-expression of PSmad3 and Sox9 in DG at P3 (arrows in **B2–B4**). **(C1–C4)** Sox9 expression in GFP+ RGLs in DG at P3 (arrows in **C2–C4**). **(D1–D4)** PSmad3+ RGLs do not co-express NeuroD (arrows in **D2–D4**). Box in panels (**A1,B1,C1,D1**) indicates region shown in panels (**A2–A6,B2–B4,C2–C4,D2–D4**). Scale bars = 200 μm in **A1,B1,C1,D1**; 50 μm in **A2–A6,B2–B4,C2–C4,D2–D4**.

Previous studies have suggested that Sox9 contributes to the maintenance stemness in a variety of tissue-specific stem cells (Furuyama et al., 2011; Guo et al., 2012), including RGLs/neural stem cells (Scott et al., 2010). Our data showed that PSmad3 was expressed in 74% ± 3% of Sox9+ cells (*n* = 4), and that Sox9 was expressed in GFP+ RGLs at P1–P6 (69.5 ± 3.2%, *n* = 9; Figures 6A1–A6,B1–B6,C1–C4). These data suggest that PSmad3 is expressed in a subpopulation of RGLs. In contrast, a vast majority of PSmad3+ RGLs did not express NeuroD, a marker for neuronal progenitors and early differentiating neurons at P3 (*n* = 6; Figures 6D1–D4). In summary, our data suggest that many PSmad3+ cells are proliferative RGLs during the first postnatal week.

**Figure 7:**
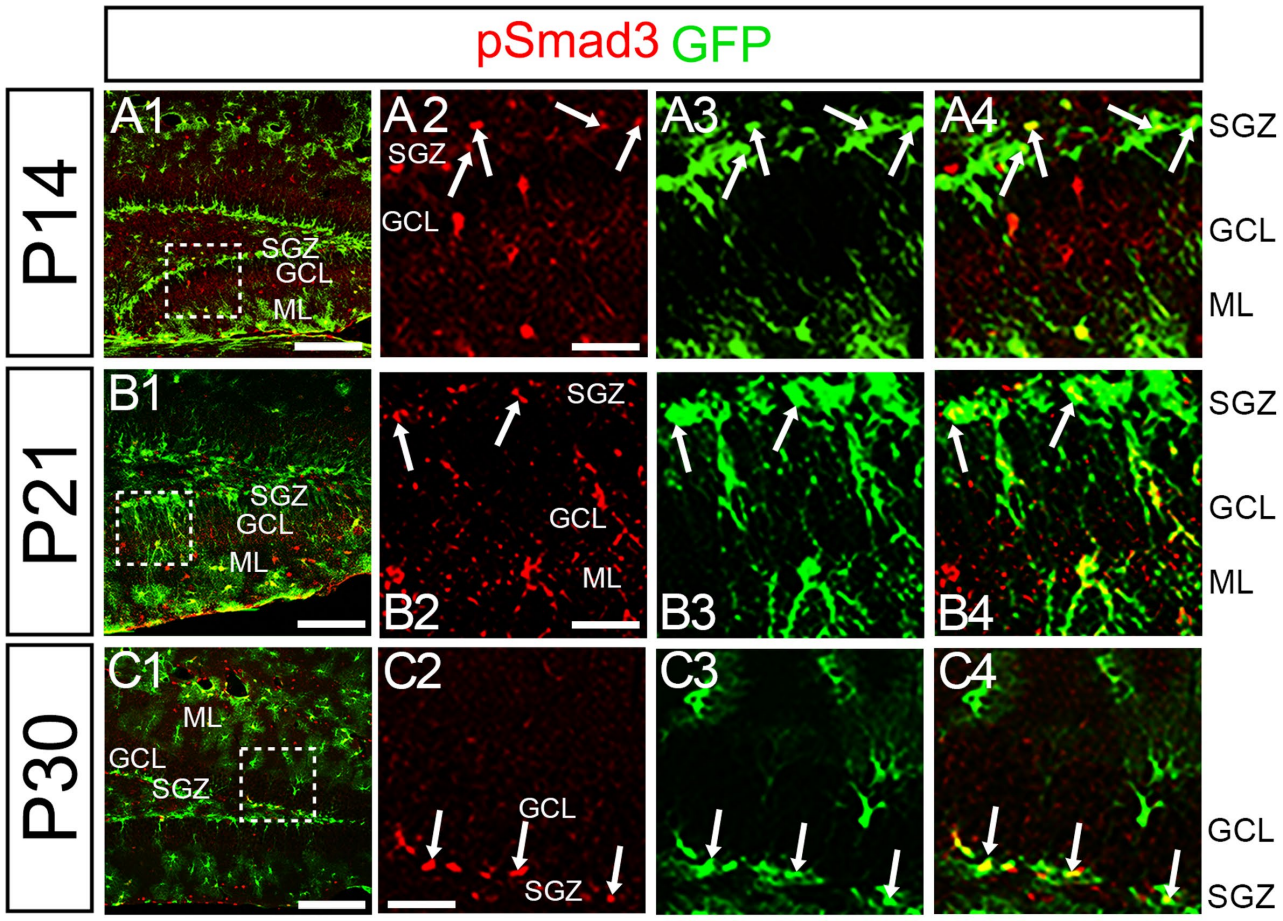
PSmad3 is expressed in GFP+ RGLs at P14 and onward **(A1–C4)** PSmad3 is expressed in GFP+ RGLs at SGZ and astrocytes in ML at P14, P21, and P30 (arrows in **A2–A4,B2–B4,C2–C4**). Box in panels (**A1,B1,C1**) indicates region shown in panels (**A2–A4,B2–B4,C2–C4**). Scale bars = 200 μm in **A1,B1,C1**; 50 μm in **A2–A4,B2–B4,C2–C4**.

**Figure 8:**
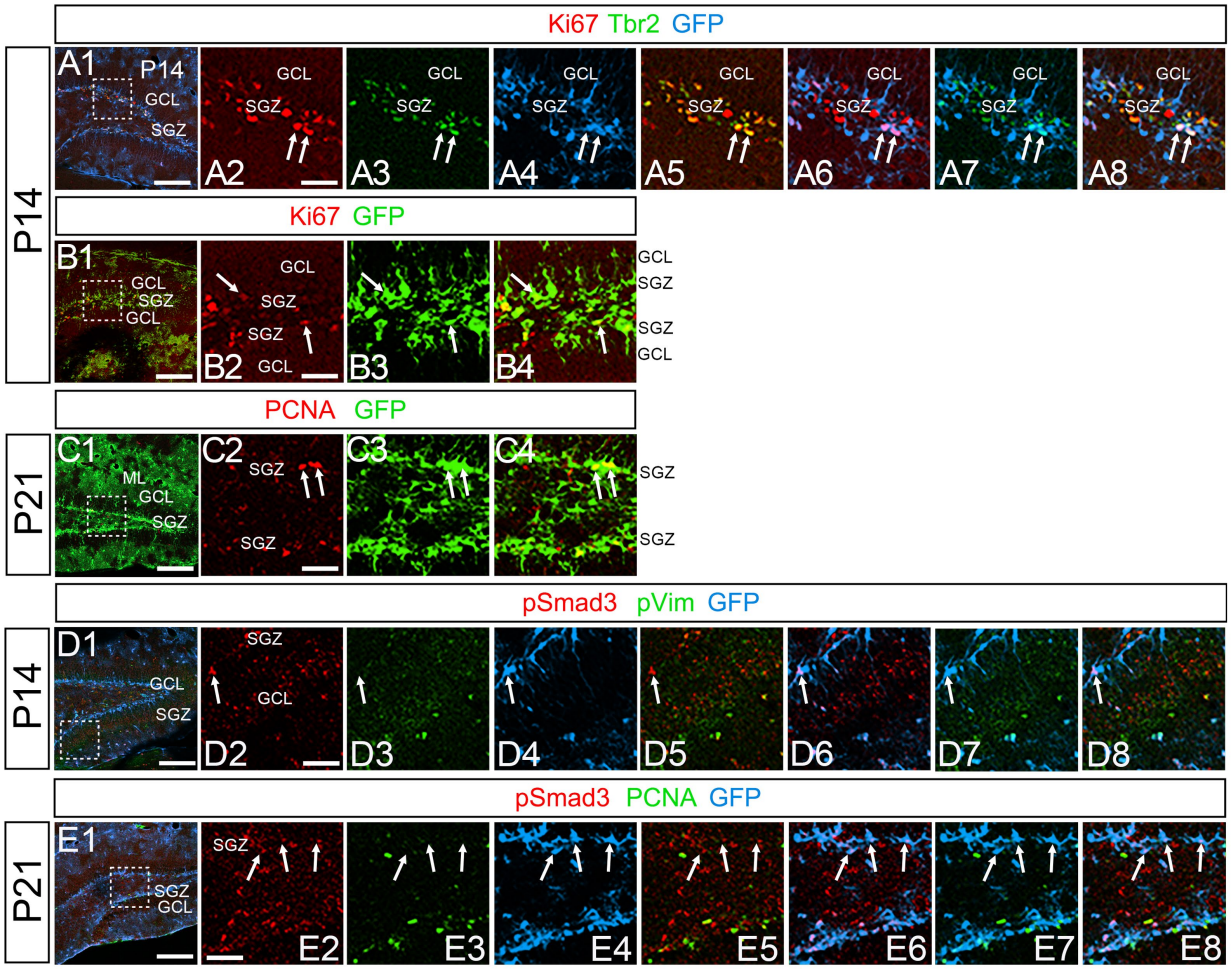
PSmad3+/GFP+ RGLs at SGZ are mostly in quiescence **(A1–A8)** Ki67 expression in Tbr2+/GFP+ progenitors at P14 (arrows in **A2–A8**). A majority of GFP+ RGLs at SGZ do not co-express Ki67. **(B1–B4)** Ki67 expression is found in some GFP+ RGLs at P14 (arrows in **B2–B4**). **(C1–C4)** PCNA expression in some GFP+ RGLs at SGZ at P21. **(D1–D8)** PSmad3+/GFP+ RGLs do not express pVim at P14 (arrows in **D2–D8**). **(E1–E8)** PSmad3+/GFP+ RGLs do not express PCNA at P21 (arrows in E2-E8). Box in panels **(A1,B1,C1,D1,E1)** indicates region shown in panels (**A2–A8,B2–B4,C2–C4,D2–D8,E2–E8**). Scale bars, 200 μm in **A1,B1,C1,D1,E1**; 50 μm in **A2–A8,B2–B4,C2–C4,D2–D8,E2–E8**.

At P14 the formation of the GCL and SGZ was almost complete (Figure 7A1). PSmad3 expression was found in the GFP+ cells at SGZ and astrocytes in the ML at P14–P30 (*n* = 3; Figures 7A1–C4). Intriguingly, many of the Ki67+ cells at the SGZ were Tbr2+ neuronal progenitors (*n* = 3; Figures 8A1–A8), and only a small proportion of the GFP+ cells were Ki67+ and PCNA+ (*n* = 3; Figures 8B1–C8). Consistent with this, PSmad3+ cells at P14-P21 were predominantly pVim−/PCNA− (Figures 8D1–E8). These data suggest that the PSmad3+ RGLs are mostly quiescent after the second postnatal week.

### Olig2 expression in PSmad3+/GFP+ astrocytes but not in RGLs at SGZ

**Figure 9:**
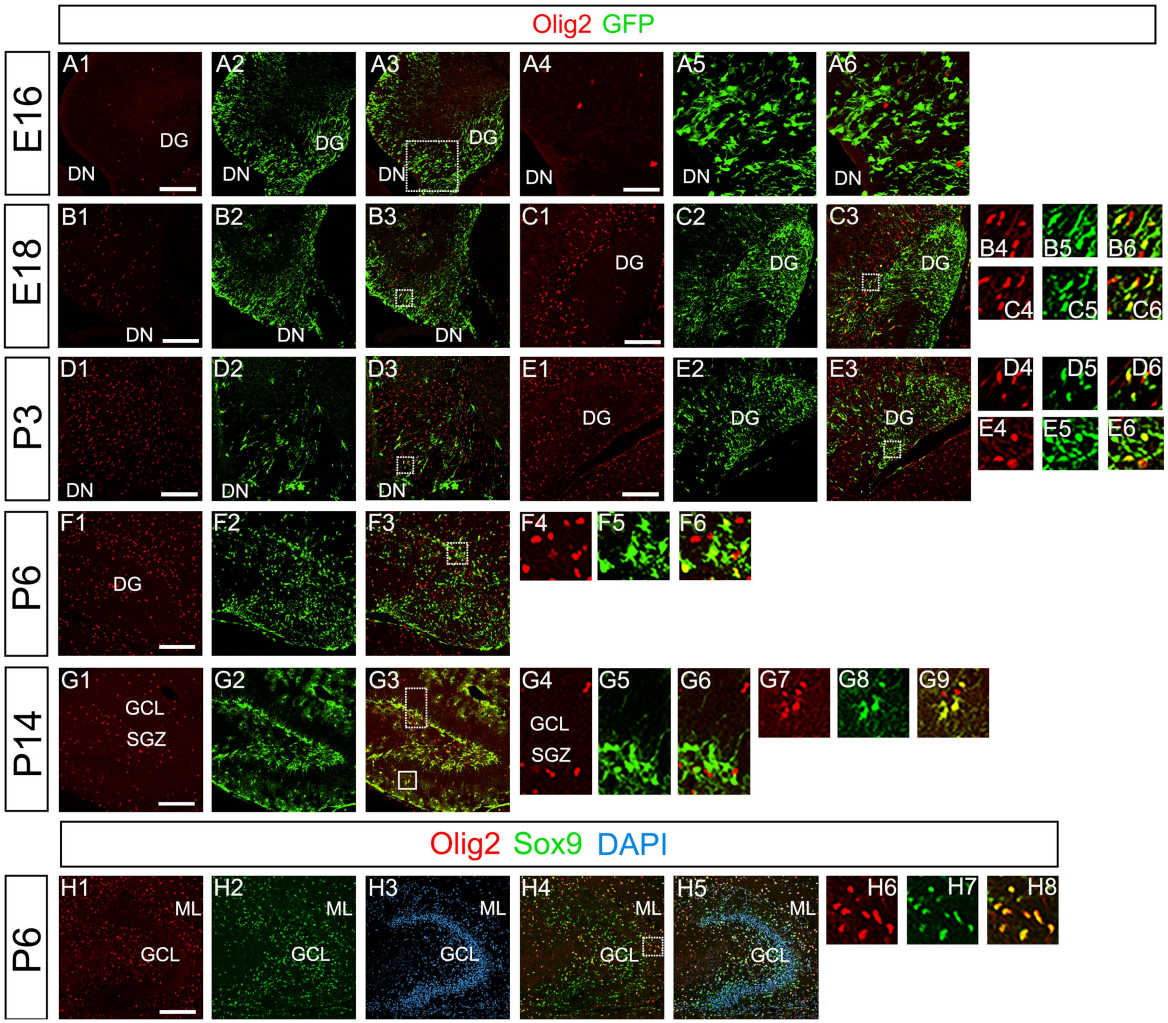
Olig2 expression in GFP+/Sox9+ astrocytes of the ML but not in the SGZ RGLs of developing mouse DG **(A1–A6)** Only a few Olig2+ cells were found in DN at E16. They are immuno-negative for GFP **(A4–A6)**. **(B1–B3,C1–C6,D1–D3,E1–E6)** Some Olig2+/GFP+ astrocytes were found both in DN and DG at E18 and P3. **(F1–F6)** Olig2+/GFP+ astrocytes are found in the ML of DG at P6. **(G1–G9)** While GFP+ cells at the SGZ do not express Olig2 **(G4–G6)**, those at the ML co-express Olig2 **(G7–G9)**, indicating that the latter are astrocytes. **(H1–H8)** Olig2+/Sox9+ cells were found in the DG at P6, suggesting that the Olig2+/GFP+/Sox9+ cells are astrocytes. Box in panels **(A3,B3,C3,D3,E3,F3,G3,H4)** indicates region shown in panels **(A4-A6, B4-B6,C4-C6,D4-D6,E4-E6,F4-F6,G4-G6,G7-G9,H6-H8)**. Scale bars, 200 μm in **A1–A3,B1–B3,C1–C3,D1–D3,E1–E3,F1–F3,G1–G3,H1–H5**; 50 μm in **A4–A6**.

**Figure 10:**
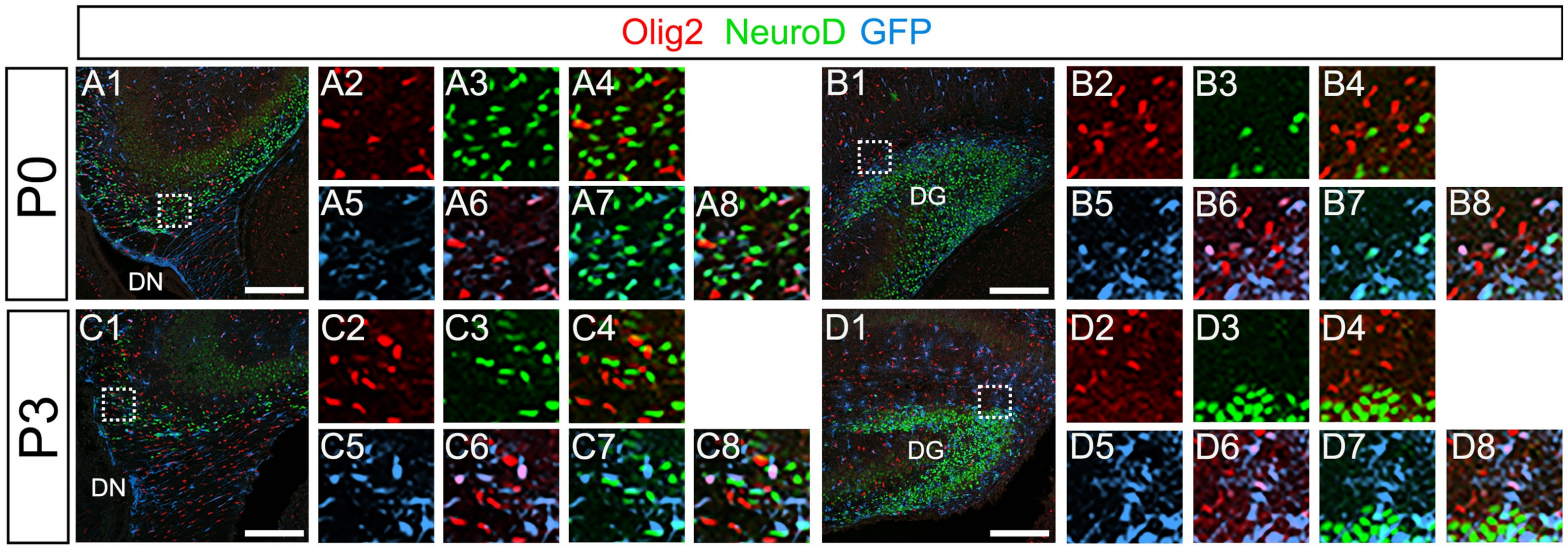
Olig2+ cells do not co-express NeuroD. **(A1–A8,B1–B8)** Olig2+/GFP+ cells do not co-express NeuroD at DN **(A1–A8)** and DG **(B1–B8)** at P0. **(C1–C8,D1–D8)** Olig2+/GFP+ cells do not co-express NeuroD at DN **(C1–C8)** and DG **(D1–D8)** at P3. Box in panels **(A1,B1,C1,D1)** indicates region shown in panels **(A2-A8,B2-B8,C2-C8,D2-D8)**. Scale bars, 200 μm in **A1,B1,C1,D1**.

Our data have shown that PSmad3 is expressed in GFP+ RGLs and astrocytes (Figures 2–7). However, the TFs expression that could discern the two distinct cell types remains unclear. Given that Olig2 is expressed not only in oligodendrocyte progenitors but also in astrocyte progenitors (Marshall et al., 2005; Zhu et al., 2012; Ohayon et al., 2018; Tatsumi et al., 2018; Wang et al., 2021), we next examined whether Olig2 is expressed in astrocytes of the developing DG. Very few Olig2+ cells were found in DG at E16 (Figures 9A1–A6). A small population of Olig2+/GFP+ cells was found in the DN and DG at E18 (Figures 9B1–B6,C1–C6). Many more Olig2+/GFP + cells were observed in both DN and DG at P3 (Figures 9D1–D6,E1–E6). 32 ± 6% of the GFP+ cells were Olig2+ (*n* = 4). The Olig2+ cells were mainly located in the ML, with some in the hilus and GCL at P3–P6 (Figures 9D1–F6). Olig2 is expressed in 42.1% ± 2.9% of Sox9+ cells (*n* = 5). GFAP is expressed in RGLs and astrocytes but not in oligodendrocyte progenitors (OLPs). Hence our data suggest that Olig2+/GFP+ cells are astrocytes. Meanwhile, Olig2+/GFP– oligodendrocyte progenitors (OLPs; 68.7% ± 4.3% of Olig2+ cells, *n* = 4) were found in the hilus, GCL, and ML (Figures 9D1–F6). At P14, Olig2+/GFP+ astrocytes were also found in the ML but not in the SGZ (Figures 9G1–G9), and they co-expressed an astrocytic marker Sox9 (Figures 9H1–H8). To confirm that Olig2 expression is associated with glial progenitors in the DG, no Olig2+ cells co-expressed a neuronal progenitor marker NeuroD (Figures 10A1–D8).

**Figure 11:**
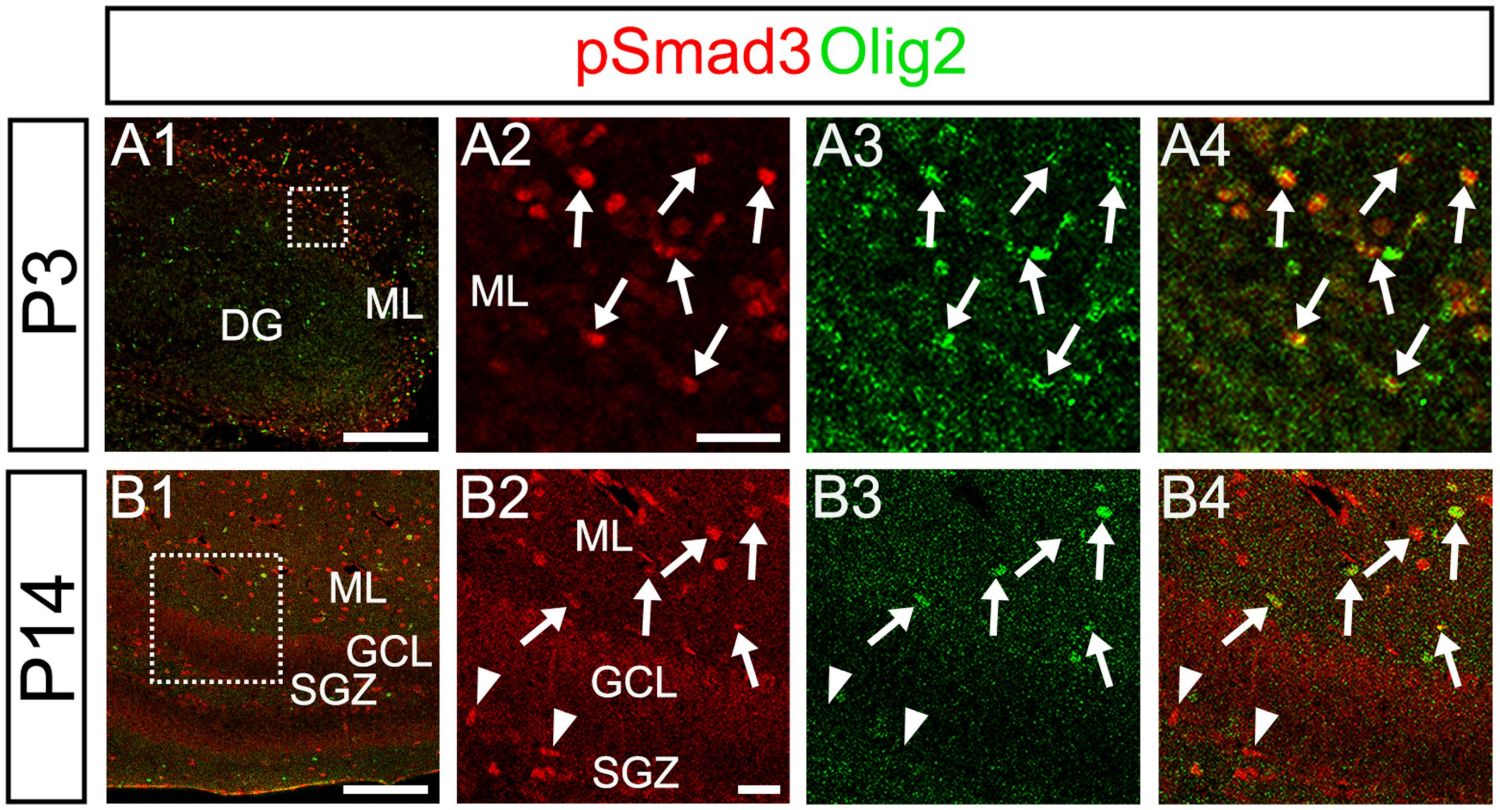
While pSmad3+ cells in the ML co-express Olig2, PSmad3+ SGZ cells do not. **(A1–A4)** PSmad3+ cells in the ML co-express Olig2 at P3 (arrows in **A2–A4**). **(B1–B4)** While PSmad3+ cells in the ML co-express Olig2 (arrows in **B2–B4**), PSmad3+ cells (red) at SGZ do not co-express Olig2 (arrowheads in **B2–B4**). Box in panels **(A1,B1)** indicates region shown in panels **(A2–A4,B2–B4)**. Scale bars, 200 μm in **A1,B1**; 50 μm in **A2–A4,B2–B4**.

**Figure 12:**
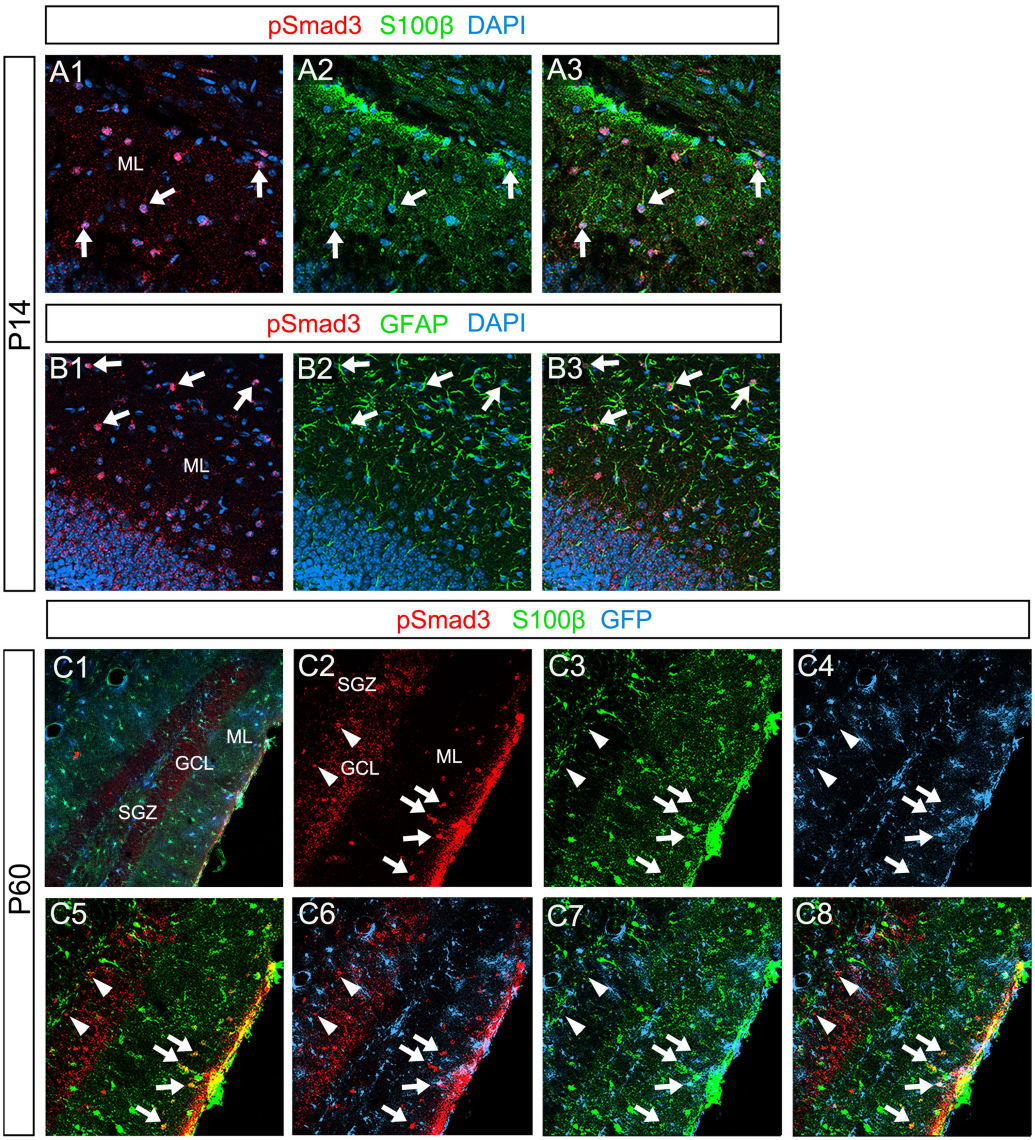
PSmad3+ cells in the ML, but not at the SGZ, co-express S100β in postnatal and adult mouse DG **(A1–A3)** PSmad3+ cells co-express S100β at ML at P14 (arrows). **(B1–B3)** PSmad3+ cells co-express GFAP at P14 (arrows). **(C1–C8)** At P60 pSmad3 is co-expressed with both S100β and GFP in the ML but not at the SGZ (arrows in **C2–C8**, arrowheads in **C2–C8**, respectively).

Our data further showed that PSmad3 is expressed in Olig2+ cells at the ML at P3 (*n* = 3, Figures 11A1–A4). At P14, PSmad3+ cells in the ML co-expressed Olig2, whereas those at the SGZ did not (*n* = 3, Figures 11B1–B4, also see Figures 9G4–G9). Collectively, our data show that PSmad3+ cells in the ML but not the SGZ of early postnatal DG are Olig2+/GFP+ astrocytes. Consistent with this, PSmad3 was expressed in S100β + and GFAP+ astrocytes in the ML at P14 (*n* = 3, Figures 12A1–A3,B1–B3, respectively). Moreover, at P60, whereas PSmad3+ cells in the ML co-expressed either S100β or GFP, PSmad3+ cells in the SGZ did not (*n* = 3, Figures 12C1–C8).

**Figure 13:**
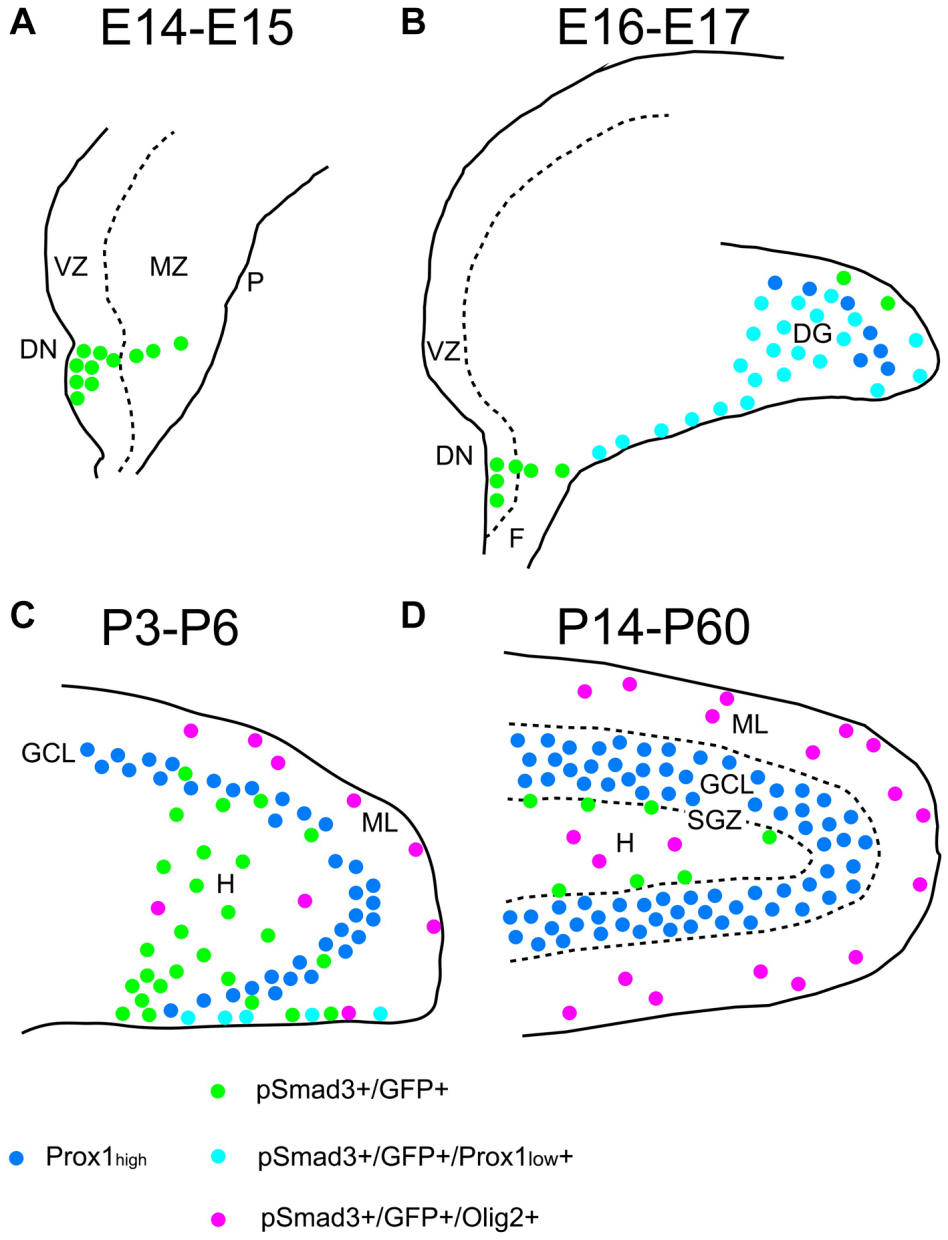
Schematic drawing of the PSmad3+/GFP+ RGLs and PSmad3+/GFP+/Olig2+ astrocytes in developing mouse DG **(A)** At E14-E15 PSmad3+/GFP+ cells (green) are located mainly at the VZ of lower DN, forming a migratory stream to the MZ. **(B)** At E16-E18 PSmad3+/GFP+/Prox1 + _low_ cells (light blue) are found in the subpial region and the DG, suggesting that they are the RGLs that give rise to granule neuronal progenitors. Very few GFP+ cells co-express Olig2. **(C)** At P3-P6 PSmad3+/GFP+ cells accumulate in the hilus (H) and ML (green, light blue, or purple). Some PSmad3+/GFP+ cells co-express either Prox1 + _low_ (blue; data not shown) or Olig2 (purple). **(D)** At P14-60 PSmad3+/GFP+ cells located at the ML and H, but not at the SGZ, co-express Olig2 (purple). Together, expressions of PSmad3 and Olig2 in combination defines RGLs (PSmad3+/Olig2− in either green or light blue) and astrocytes (PSmad3+/Olig2+ in purple) both in embryonic and postnatal DG. DG, dentate gyrus; DN, dentate notch; F, fimbria; GCL, granule cell layer (Prox1_high_ + in blue); H, hilus; MZ, mantle zone; P, pia; VZ, ventricular zone.

Taken together, GFP+ and Sox9+ cells are either RGLs or astrocytes in the postnatal DG. Approximately 60%–70% of them correspond to RGLs (Sox9 + Olig2– or GFP+ Olig2–). The remaining 30%–40% are astrocytes (Olig2 + GFP+ or Olig2 + Sox9+). Approximately 70% of the Sox9+ cells (RGLs or astrocytes) co-express PSmad3. Approximately 30% of the GFP^+^ cells (RGLs or astrocytes) are proliferating (Ki67+) at during the first postnatal week. In conclusion, the present study has revealed that there are distinct types of PSmad3+/GFP+ cells in both embryonic and postnatal mouse DG: neural progenitors, RGLs, and Olig2+ astrocytes (Figure 13).

## Discussion

### Development of PSmad3+ RGLs

*Gfap-*GFP+ (GFP+) DG progenitors express Sox2 and migrate away from the VZ of the DN, and they are found in the SGZ, ML, and hilus in the postnatal DG (Figure 1). These data suggest that both the GFP+ RGLs in the SGZ and the astrocytes in the ML and hilus are derived from the DN. Consistent with this, PSmad3 expression was found in both GFP+/Prox1 + _low_ progenitors, GFP+ RGLs, and GFP+/Olig2+ astrocytes. Given the Prox1_low_ + expression, the PSmad3+/ GFP+ progenitors seem to possess a property of neurogenic cells that give rise to Prox1_high_ + dentate granule neurons. Interestingly, PSmad3+/GFP+/Prox1_low_ + progenitors were also observed in early postnatal DG (data not shown). However, the developmental origin of PSmad3+ RGLs and astrocytes remains elusive. Given that PSmad3+ cells are observed mostly in the lower DN and fimbria-dentate junction at E14–17, the PSmad3+ RGLs are likely to originate from the lower DN. Consistent with this, PSmad3 is expressed in a subpopulation of GFP+/Sox9+ cells that are likely to originate from a broader region around the DN (Figure 6). While our data imply that PSmad3+/ GFP+/Prox1 _low_ + neurogenic progenitors may originate from the lower DN, a genetic lineage tracing analysis will be necessary to further clarify this issue.

It has previously been shown that expansion of RGLs is prominent during the first week after birth (Matsue et al., 2017). In support of this notion, the expansion of PSmad3+/GFP*+* RGLs becomes evident during the perinatal period (Figure 5; Supplementary Figure 3). The majority of the PSmad3+ RGLs co-express Sox2, Sox9, pVim, and PCNA (Figures 3, 5, 6; Supplementary Figure 3), supporting their proliferative capacity in the first postnatal week.

The TGFβ-pSmad2/3-Sox2 pathway has been implicated in regulating the stemness of glioma-initiating cells (Ikushima et al., 2009). Recent studies have also shown that PSmad3 can bind to Sox9 and activate Sox9-dependent transcription (Furumatsu et al., 2009), and that Sox9 and Slug co-operate to maintain cancer stem cells (Guo et al., 2012). While Slug is not expressed in the developing brain (Marin and Nieto, 2004), Snail1, a cousin of Slug controls the number of neural progenitors at the SGZ of the hippocampus (Zander et al., 2014). Similarly, Zeb1 at the downstream of PSmad3 maintains RGLs in the DG (Gupta et al., 2021). PSmad3 may contribute to control the proliferative property of RGLs in the DG through the regulation of the EMT-TFs Sox9, Snail1, and Zeb1.

Sufu acts as a positive regulator of Shh signal, thereby RGLs expansion in the DG (Noguchi et al., 2019). TGFβ2 induces Gli1 expression in a PSmad3-dependent manner after ischemia/reperfusion injury (Peng et al., 2019). Future analysis of the crosstalk between the TGFb-PSmad3 pathway and Shh signaling will also shed light on the mechanism controlling the expansion of postnatal RGLs in the DG.

Accumulating evidence suggests that EMT prevents oncogene-induced senescence (OIS) early in tumor progression (Weinberg, 2008). EMT TFs such as Twist and Zeb1 promote EMT and simultaneously inhibit OIS (Ansieau et al., 2008; Chaffer et al., 2013). Deficiency of the senescence regulator p21^CIP1^ induces EMT vice versa (Lin et al., 2012). Β-catenin associates with PSmad3 to promote TGFβ-induced EMT (Tian et al., 2012). In contrast, downregulation of Wnt-β-catenin signaling triggers the onset of cellular senescence (Ye et al., 2007). Thus, it is possible that TGFβ and Wnt signaling may cooperate at the level of PSmad3 and β-catenin to escape senescence in RGLs, thereby maintaining their stemness.

Tapia-Gonzalez et al. showed that Smad3 is expressed in early intermediate progenitors but not in Sox2+ RGLs in adult mouse DG. Smad3 mRNA was observed in the granule cell layer (GCL) in addition to the SGZ. Our data show that PSmad3 is expressed in GFP+/Sox2+/Sox9+ progenitors and RGLs. These data appear to be somewhat contradictory each other. Tapia-Gonzalez et al. analyzed the DG of 3–4 month old female mice. In our data, PSmad3 expression was observed in the SGZ, hilus, and ML in male mice at P60 (Figure 12). It is also worth noting that PSmad3 was very weakly expressed in the GCL of the mouse DG at P60 but not at P30 (Figures 7, 12). Intriguingly, at 6 months old of age, PSmad3 was strongly expressed in the GCL in addition to the SGZ, hilus, and ML (data not shown). It seems that the expression pattern of PSmad3 is somehow slightly different between adolescent and adult mouse DG.

Our data clearly show that PSmad3 is expressed in subpopulation of GFP+ cells. This also explain why in Tapia-Gonzalez et al., PSmad3 expression was not observed in Sox2+ and GFAP+ RGLs at the SGZ of 3–4 month old mouse DG (Tapia-González et al., 2013). GFP+ expression can label many more GFAP-expressing cells, compared to immunolabeling with GFAP antibody. Given our data that some GFP*+* cells at the SGZ express Tbr2 (unpublished data), and that Tbr2+ intermediate progenitors at the SGZ express Ascl1 (Tapia-González et al., 2013), this may explain why we were able to detect PSmad3+/GFP*+* RGLs at the SGZ, whereas PSmad3 expression was detected in Ascl1+ progenitors (Tapia-González et al., 2013).

Taken together, PSmad3 is expressed in a subpopulation of GFP*+*/Sox2+/Sox9+ dentate progenitors in the embryo, and the RGLs and astrocytes in the postnatally developing mouse DG. Although this also seems to be the case in the adult, further careful analysis of PSmad3 expression in combination with various markers of distinct cell types is necessary to accurately describe the expression pattern of PSmad3 throughout life.

### Development of PSmad3+/Olig2+ astrocytes

While PSmad3+ progenitors were found in the DN at E14–E16, very few Olig2+ cells were found in both the DN and DG at E16 (Figures 1, 2, 9). Olig2+ progenitors appear at the upper DN at E18 (Figures 9B1–B6). At P1-6 PSmad3+/GFP*+* progenitors were found around both upper and lower DN, and many of them co-expressed Olig2 (data not shown). These data suggest that the Olig2+ astrocytes seem to originate from the VZ around the DN from E18 onwards.

Recent studies have shown that Olig2+ astrocytes are present in both embryonic and adult CNS (Ohayon et al., 2018; Tatsumi et al., 2018; Wang et al., 2021). While it has previously been shown that the majority of Olig2+ astrocytes are GFAP− in the adult CNS (Tatsumi et al., 2018; Wang et al., 2021), we were able to detect Olig2+/GFP+ astrocytes by using *gfap-*GFP mice (Figure 9). Nonetheless, there may be Olig2+/GFP– astrocytes in the DG. More careful analysis of Olig2+ astrocytes will be necessary to clarify the heterogeneity of Olig2+ astrocytes. In terms of the function of Olig2+ astrocytes, while Olig2+ OLPs respond to brain injury such as hypoxia and increase in number, the role of Olig2+ astrocytes remains unclear (Allan et al., 2021). It will be interesting to see whether Olig2+ astrocytes act as reactive astrocytes after brain injury.

In conclusion, we have provided evidence that PSmad3 is expressed in a subpopulation of GFP*+*/Sox9+ neural progenitors, RGLs, and astrocytes in the mouse DG during embryonic and postnatal development, and that Olig2 expression is allocated with astrocytes but not RGLs. While the roles of PSmad3 and Olig2 in the development of RGLs and astrocytes remain elusive, combinatorial expression of these TFs will be useful to identify RGLs and Olig2+ astrocytes to investigate their development and function in the mouse DG.

## Data availability statement

The original contributions presented in the study are included in the article/Supplementary material, further inquiries can be directed to the corresponding author.

## Ethics statement

The animal study was approved by Ethics committee of Animal Experiments in Tokyo Medical University. The study was conducted in accordance with the local legislation and institutional requirements.

## Author contributions

KO: experimental design. KO, SO, TK, TS, and KT: acquisition of data. KO and HS: analysis and interpretation of data and writing and editing the manuscript. All authors contributed to the article and approved the submitted version.

## Funding

This study was supported by Mitsui Sumitomo Insurance Welfare Foundation (to KO), Japan Society for the Promotion of Science (JSPS) KAKENHI [Grant numbers 19 K11726 (to KO), 22H03367 (to KO), 23 K05995 (to KO), 16 K18983 (to HS), and 20 K07233 (to HS)], the Center for Diversity at Tokyo Medical University TMUCD-202202 (to HS), and Research promotion grant from Tokyo Medical University (to KO).

## Conflict of interest

The authors declare that the research was conducted in the absence of any commercial or financial relationships that could be construed as a potential conflict of interest.

## Publisher’s note

All claims expressed in this article are solely those of the authors and do not necessarily represent those of their affiliated organizations, or those of the publisher, the editors and the reviewers. Any product that may be evaluated in this article, or claim that may be made by its manufacturer, is not guaranteed or endorsed by the publisher.
