# Supplementary figures and images for: PSmad3+/Olig2− expression defines a subpopulation of gfap-GFP+/Sox9+ neural progenitors and radial glia-like cells in mouse dentate gyrus through embryonic and postnatal development
Source: Front Neurosci. 2023 Sep 18;17:1204012. doi: 10.3389/fnins.2023.1204012 (PMC10547214; doi:10.3389/fnins.2023.1204012)

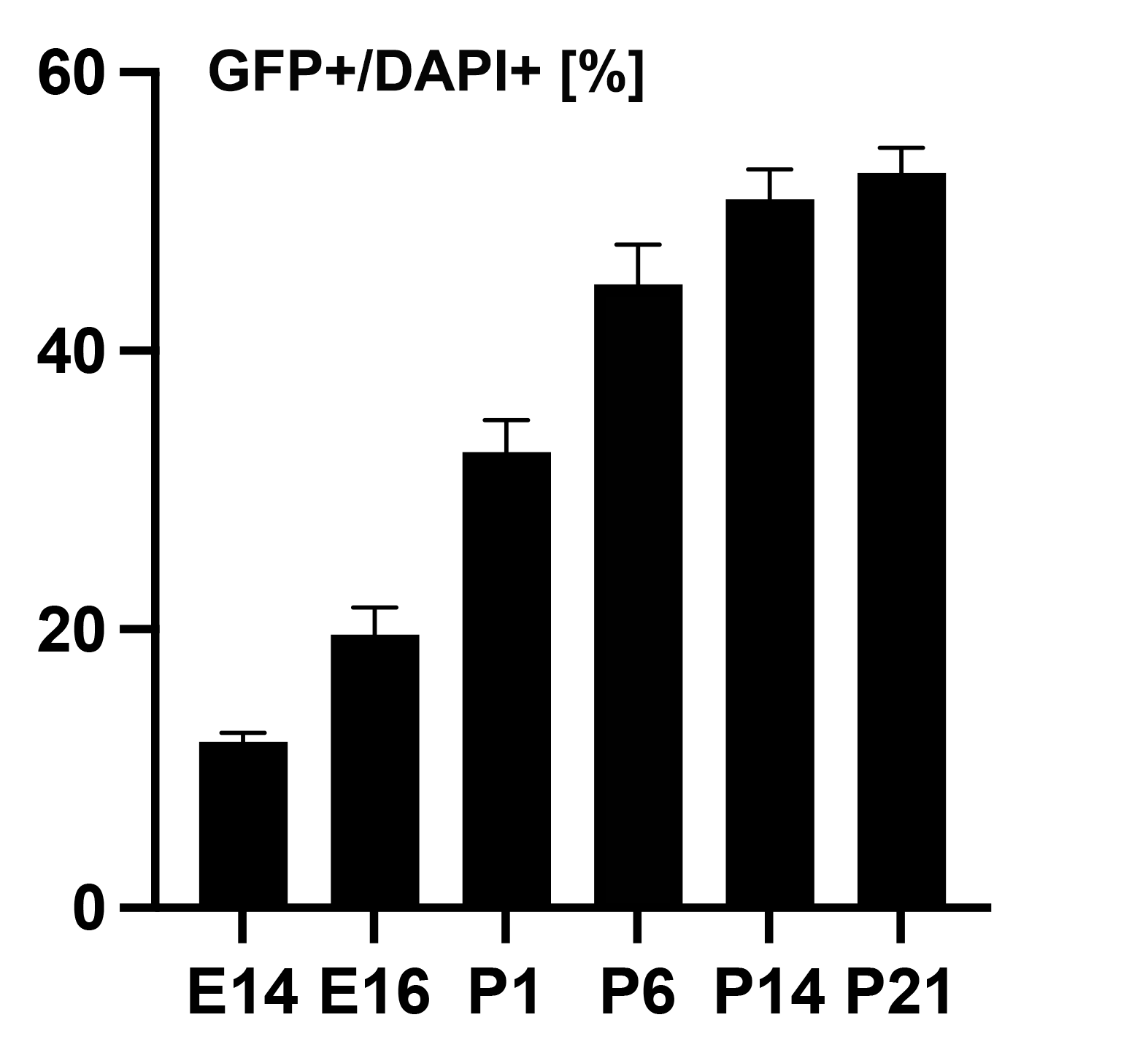

Supplement: Supplementary file 1 [file Image_1.TIF]

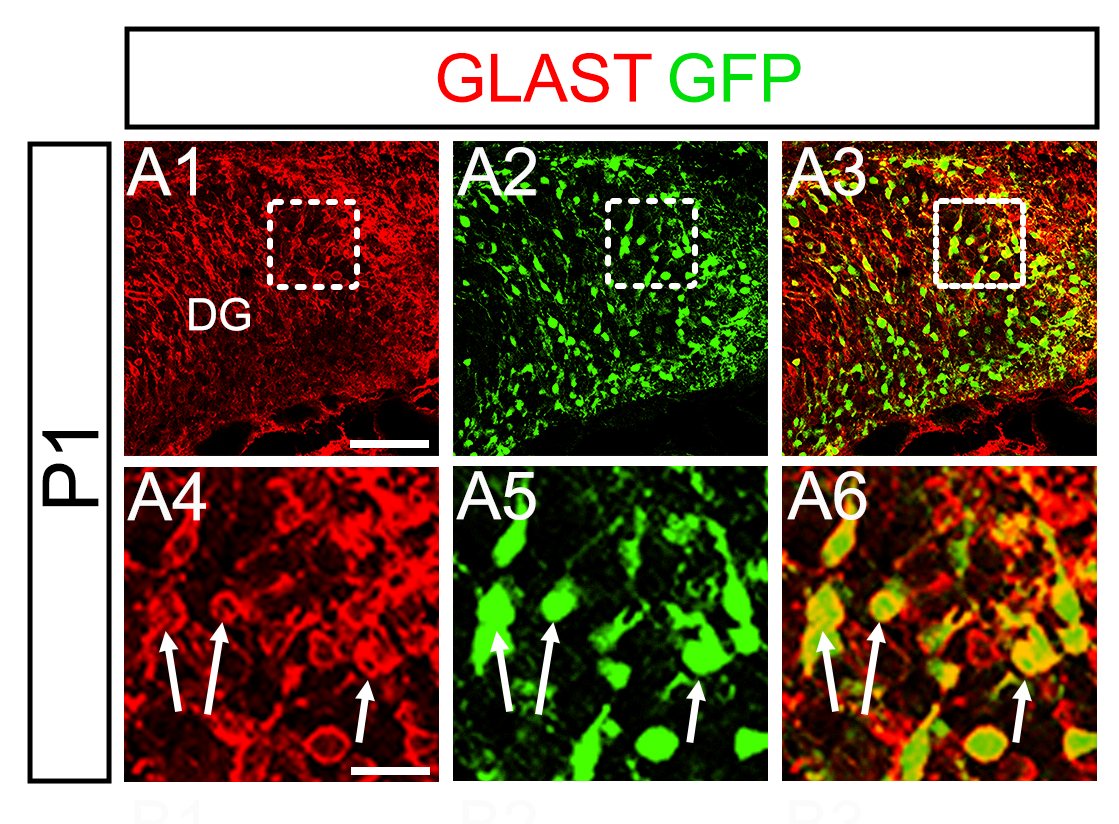

Supplement: Supplementary file 2 [file Image_2.TIF]

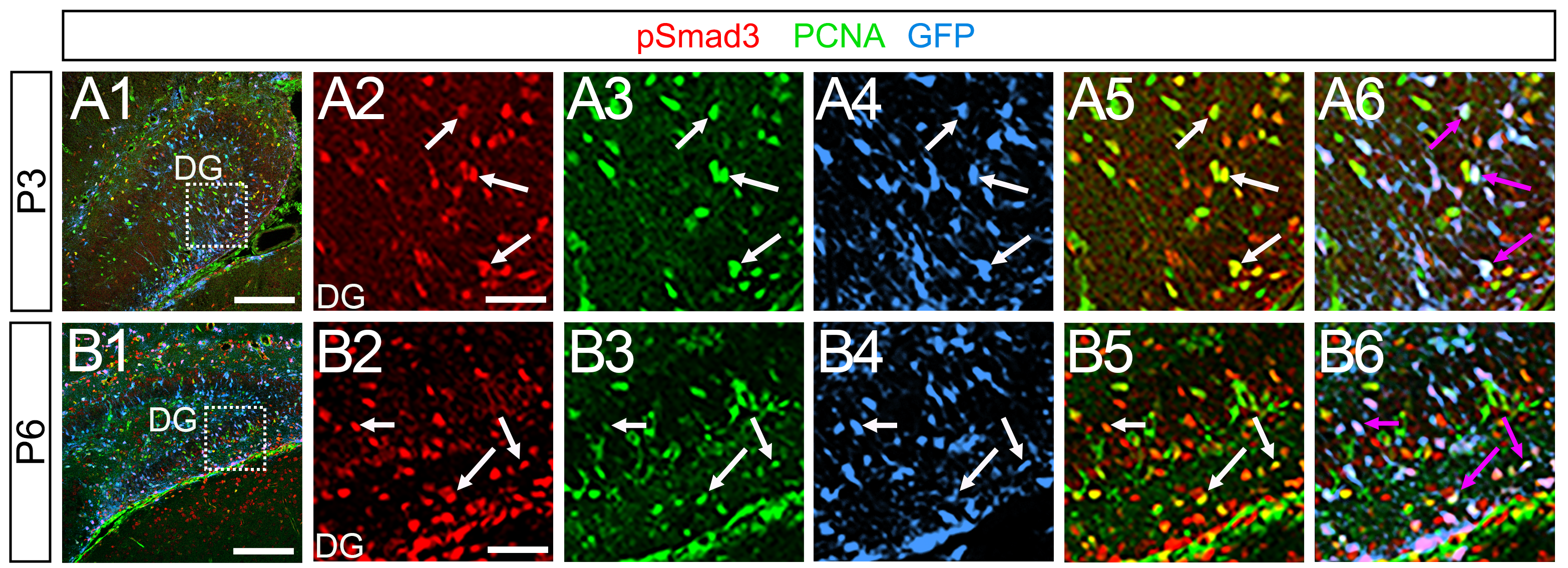

Supplement: Supplementary file 3 [file Image_3.TIF]

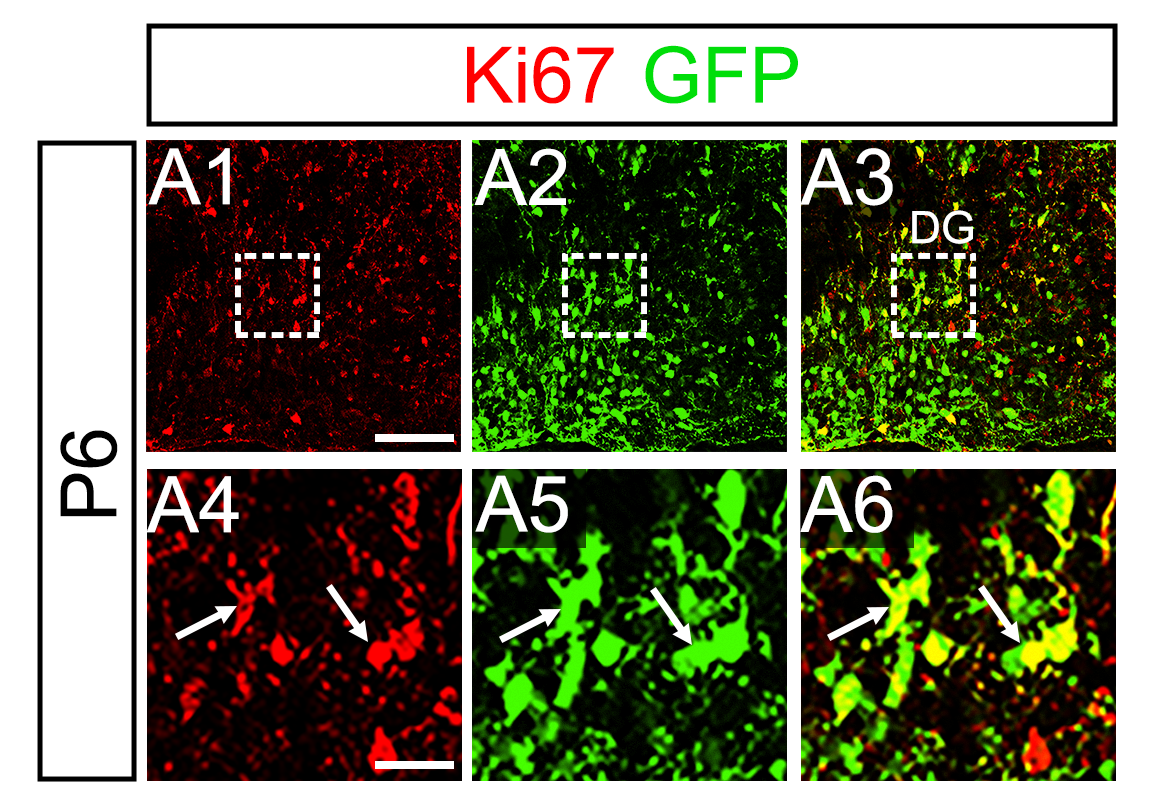

Supplement: Supplementary file 4 [file Image_4.TIF]
